# A Case of Small-Cell Lung Cancer With Novel Anaplastic Lymphoma Kinase Gene Rearrangement That Developed Intradural Extramedullary Spinal Metastases With Myelitis

**DOI:** 10.7759/cureus.75369

**Published:** 2024-12-09

**Authors:** Yuri Mizuno, Yuji Tada, Taira Uehara, Satoshi Yamashita, Hiroyuki Murai

**Affiliations:** 1 Department of Neurology, International University of Health and Welfare Narita Hospital, Narita, JPN; 2 Department of Respirology, International University of Health and Welfare Narita Hospital, Narita, JPN; 3 Epilepsy and Sleep Center, Fukuoka Sanno Hospital, Fukuoka, JPN

**Keywords:** alectinib, anaplastic lymphoma kinase gene, myelitis, small-cell lung cancer, spinal metastasis

## Abstract

*Anaplastic lymphoma kinase* (*ALK)* gene rearrangement-positive small-cell lung cancer (SCLC) is extremely rare. A 73-year-old man was diagnosed with SCLC. Standard treatments were not effective. Furthermore, at 74 years of age, intradural extramedullary metastases in the lumbar spinal cord and myelitis were observed. Autoimmune myelitis was suspected because anti-Zic4 antibodies were detected. However, steroid pulse therapy was ineffective. Interestingly, a novel *ALK* rearrangement of the* isoamyl acetate hydrolyzing esterase 1 (IAH1)-ALK *fusion gene was identified by blood-based next-generation sequencing. Although it was unclear whether the *IAH1-ALK* fusion gene was involved in tumor progression or an asymptomatic mutation, we treated the patient with alectinib, an ALK inhibitor; however, this therapy did not reduce the lesions. There is no established effective treatment for patients with SCLC who are *ALK* fusion gene positive by liquid biopsy. Therefore, patient-specific approaches and treatments are required.

## Introduction

*Anaplastic lymphoma kinase (ALK)* gene rearrangement in lung cancer was first reported as the *echinoderm microtubule-associated protein-like 4 (EML4)-ALK* fusion gene in adenocarcinoma [1,2]. The fusion of the *ALK* gene with another gene leads to the production of an *ALK* fusion protein with abnormal activity. Since then, various *ALK* fusion partners have been discovered [3], and treatment with ALK inhibitors is noticeably effective for *ALK* fusion gene-positive lung cancer [4].

However, *ALK* rearrangements have been identified in approximately 5% of lung cancer patients, almost all of which are non-small-cell lung cancer (NSCLC) [3]. This mutation is extremely rare in small-cell lung cancer (SCLC) [5-8]; therefore, the clinical features, complications, and treatment options of SCLC with *ALK* rearrangement remain unclear. Here, we describe a rare case of SCLC with an *isoamyl acetate hydrolyzing esterase 1 (IAH1)-ALK *fusion gene, which is a novel gene mutation. This case is also remarkable because the patient developed spinal metastasis with myelitis, a type of spinal inflammation.

## Case presentation

**Figure 1:**
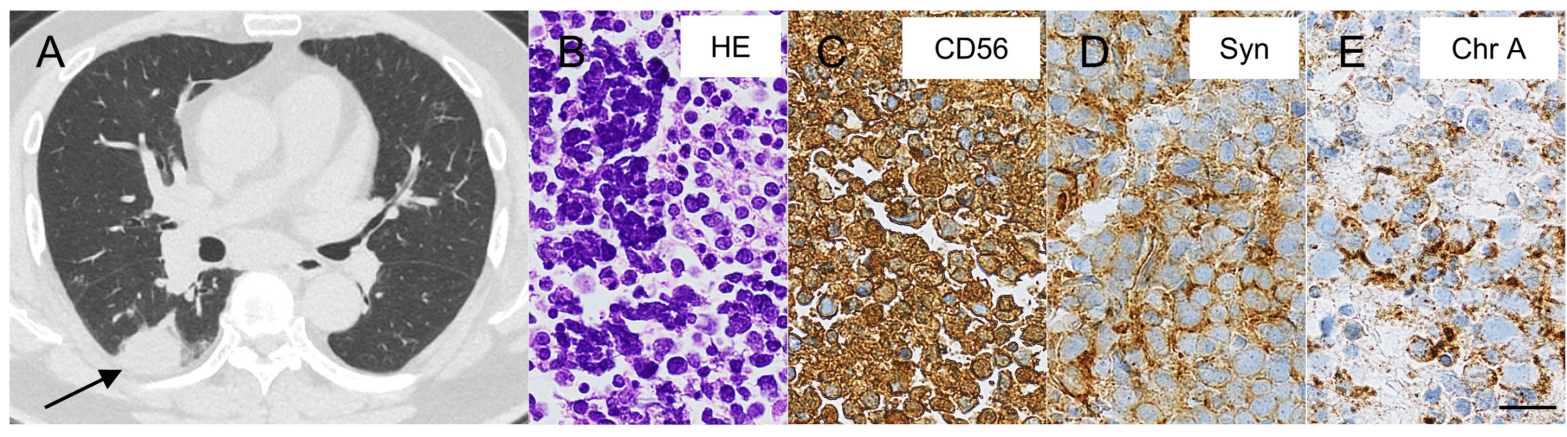
Primary small-cell lung tumor and metastases. A: computed tomography (CT) scan shows a solid lesion in the right lobe of the lung (arrow); B: hematoxylin-eosin (HE) staining of the mediastinal lymph nodes; C–E: immunohistochemistry staining of the mediastinal lymph nodes. Immunoreactivity of CD56, synaptophysin (Syn), and chromogranin A (Chr A) are detected. Scale bar = 20 μm.

A 73-year-old male ex-smoker of 30 pack-years was referred to our hospital because of fatigue and a cough. Chest computed tomography (CT) imaging revealed an approximately 3 cm diameter mass in the lower lobe of the right lung (Figure 1A) and enlarged peribronchial lymph nodes. An 18F-fluorodeoxyglucose positron emission tomography (PET)/CT scan demonstrated abnormal uptake in the right lung mass and multiple lymph nodes around the bronchi. Tumor marker values in the blood were as follows: pro-gastrin-releasing peptide 1820 pg/ml (< 81 pg/ml), neuron-specific enolase 39.1 ng/ml (< 16.3 ng/ml), carcinoembryonic antigen 5.4 ng/ml (< 5.0 ng/ml), and cytokeratin fragment 2.8 ng/ml (< 2.8 ng/ml). Samples obtained from mediastinal lymph nodes by endobronchial ultrasound-guided transbronchial needle aspiration showed tumor cells with a naked nucleus (Figure 1B). It also showed abundant immunoreactivity for CD56 (Figure 1C), synaptophysin (Figure 1D), chromogranin A (Figure 1E), and Ki-67 (approximately 90% positive); weak immunoreactivity for thyroid transcription factor 1; and negative immunoreactivity for CD20. He was diagnosed with stage IIIA (T2aN2M0) SCLC.

**Figure 2:**
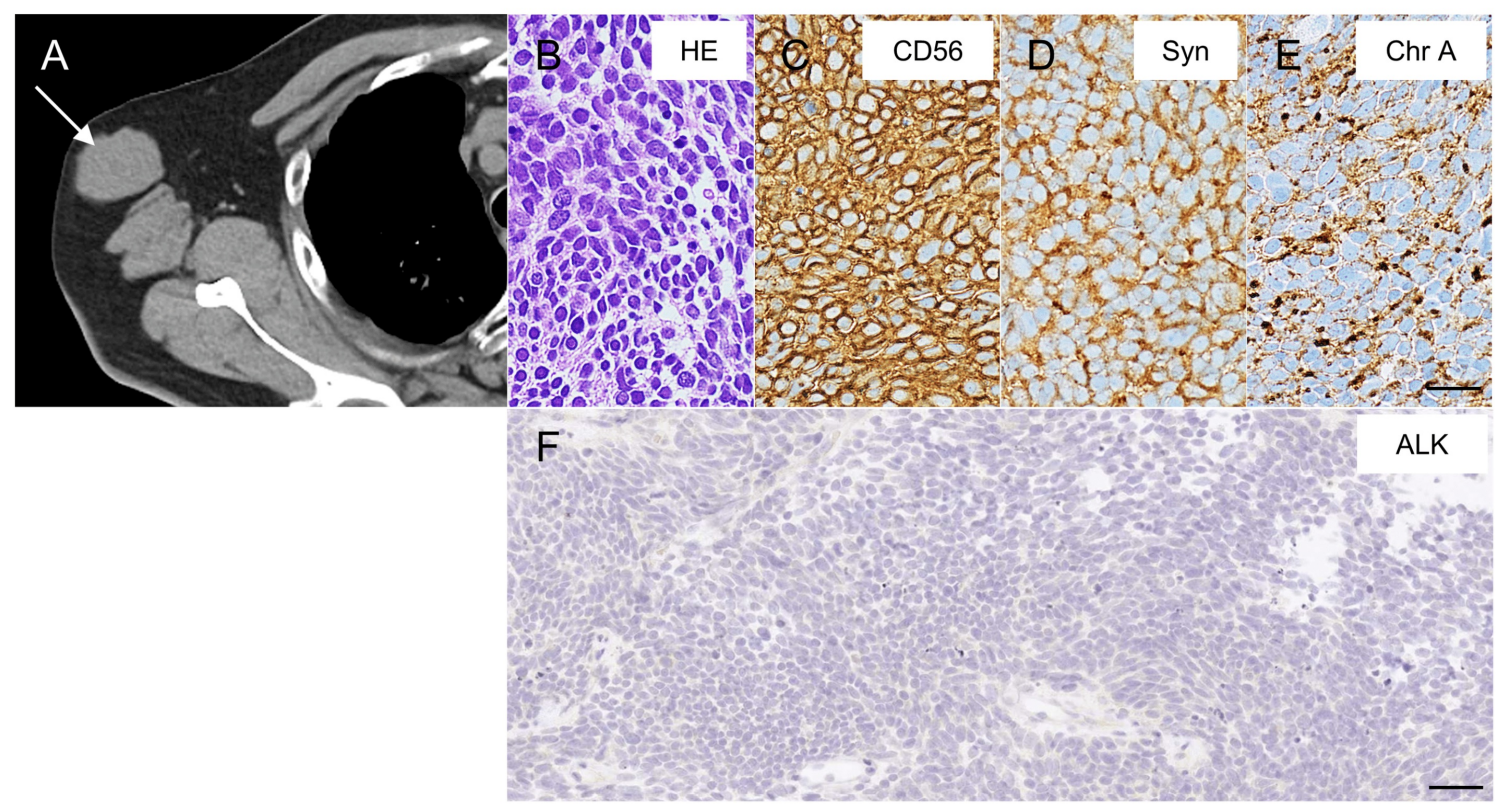
CT image and pathological images of metastases. A: CT scan shows right axillary lymph node metastases (arrow); B: hematoxylin-eosin (HE) staining of the right axillary lymph node; C–E: immunohistochemistry staining of the right axillary lymph node (immunoreactivity of CD56, synaptophysin (Syn), and chromogranin A (Chr A) are detected and Scale bar = 20 μm); F: immunohistochemistry of the anaplastic lymphoma kinase (ALK) expression in the right axillary lymph node. No signal was observed, indicating a lack of AKL-positive cells in the lymph node metastases that developed subsequently. Scale bar = 40 μm.

The patient started treatment with four cycles of combination chemotherapy consisting of cisplatin and etoposide, and thoracic radiation concurrent with the first cycle of chemotherapy (45 Gy in 30 twice-daily fractions). Initially, he had a partial response to first-line chemotherapy. However, at 74 years of age, a PET/CT scan detected subcutaneous metastases in the left buttock, right axillary lymph node metastases (Figure 2A), multiple liver metastases, and bilateral adrenal metastases. Needle biopsy from the right axillary lymph node showed tumor cells with a high N/C ratio (Figure 2B). The tumor cells had the same immunoreactivity (Figures 2C-2E) with the peribronchial lymph nodes. The second treatment consisted of combination chemotherapy with carboplatin and etoposide for one month. However, after three months of the second treatment, neither the primary lung tumor nor metastases had diminished, and he developed acute spinal cord symptoms during the same period. Therefore, he was admitted to the neurology department.

**Figure 3:**
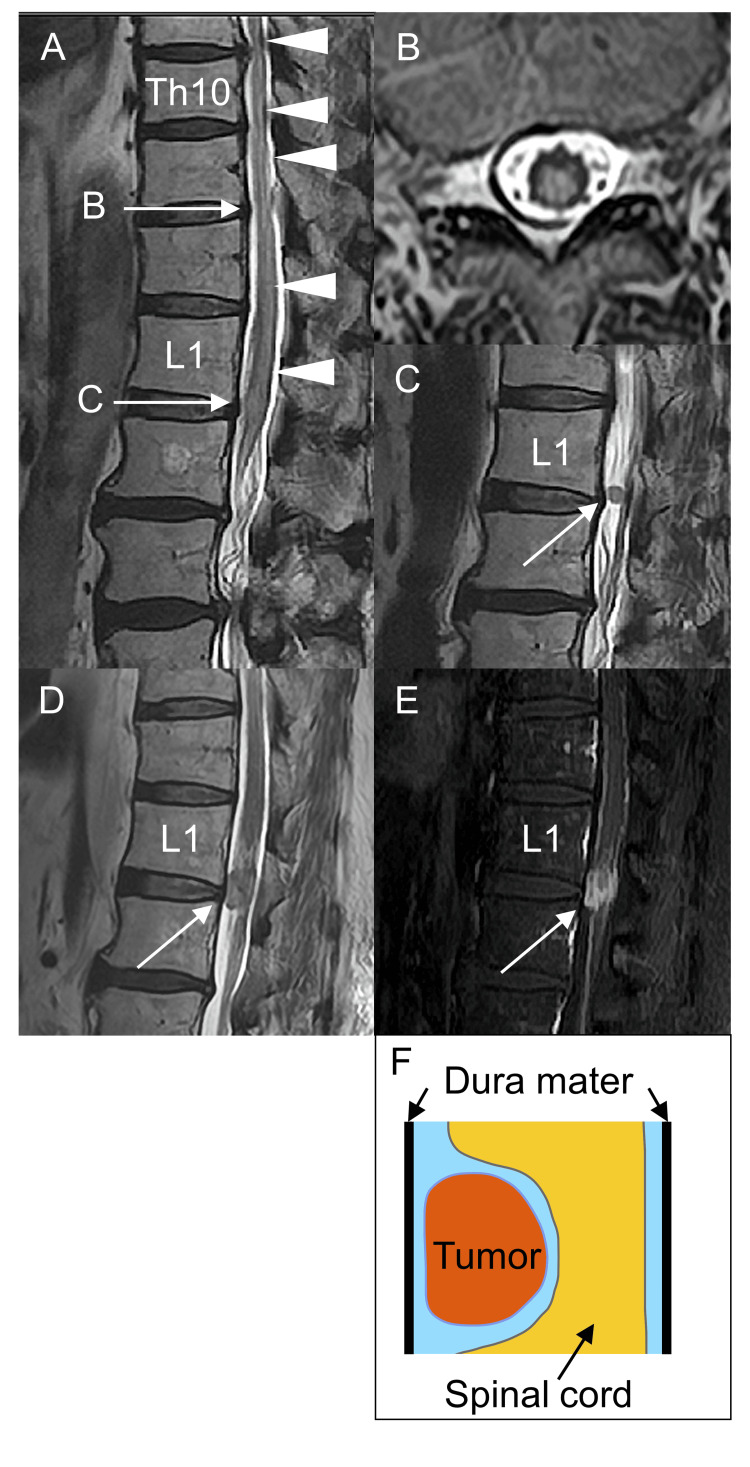
Intradural extramedullary spinal metastases with myelitis. A: sagittal T2-weighted magnetic resonance image (MRI) shows an intramedullary long spinal cord lesion from Th10 to L2 spine (arrowheads); B: axial T2-weighted image at the Th11 level shows widespread high-intensity signals in the spinal cord; C: sagittal T2-weighted image at the L1 level shows an intradural extramedullary lesion slightly to the right of center (arrow); D: despite treatment with alectinib, the intradural extramedullary metastasis lesion at the L1 level became enlarged (arrow); E: gadolinium-enhanced T1-weighted MRI shows a growing intradural extramedullary nodule (arrow); F: representation of intradural extramedullary spinal metastases.

A neurological examination at the time of admission revealed that he had mild weakness in both lower limbs. Additionally, surface sensations below the iliac crest were reduced, and deep sensations in the lower limbs were severely impaired. He could not stand by himself. His deep tendon reflexes were not increased, which might have been related to the chemotherapy. Autonomic dysfunctions, including constipation and dysuria, were also observed. Magnetic resonance imaging showed a hyperintense lesion from Th10 to L2 spine on a T2-weighted image, which suggested longitudinal extensive transverse myelitis (Figure 3A, 3B). Furthermore, there was a well-defined extramedullary lesion approximately 5 mm in diameter at the L1 spinal cord, considered at that time to be schwannoma (Figure 3C). Cerebrospinal fluid examination revealed a leukocyte count of 4 cells/μl (0-5 cells/μl), protein of 101 mg/dl (10-35 mg/dl), and negative for oligoclonal bands and malignant cells. Anti-aquaporin 4 antibodies, anti-myelin oligodendrocyte glycoprotein antibodies, anti-voltage-gated calcium channel antibodies, and anti-glutamic acid decarboxylase antibodies were negative in blood tests. Paraneoplastic myelopathy was suspected because anti-nuclear and paraneoplastic antineuronal antibodies, including anti-Zic4 and anti-recoverin antibodies, were positive [9,10]. Three courses of high-dose intravenous methylprednisolone treatment were performed; however, the weakness and sensory loss in the lower limbs did not improve.

To identify the next treatment options for the patient, we analyzed cancer-related genes by blood-based next-generation sequencing using the Foundation One® Liquid CDx assay (Chugai Pharmaceutical Co., Ltd., Tokyo, Japan). Interestingly, we detected an* IAH1-ALK* fusion gene (variant allele frequency 0.39%, tumor fraction 45%), which suggests ALK inhibitors were applicable to his treatment. However, the use of ALK inhibitors without sufficient consideration is controversial, because *IAH1-ALK *is a novel *ALK* fusion gene, and the *ALK* signal was negative as determined by the immunostaining of lymph node metastasis samples, suggesting primary tumor heterogeneity (Figure 2F). Furthermore, the number of *ALK* rearrangement-positive cells in the primary lung lesions was unclear when assessed by liquid biopsy. However, considering that standard treatment did not prevent the progression of his SCLC, we decided to treat the patient with the ALK inhibitor alectinib after careful consideration. At seven months after recurrence, the patient was treated with alectinib for approximately two months. Eventually, the extramedullary lesion at the L1 spinal level expanded rapidly with contrast enhancement after alectinib treatment (Figures 3D, 3E) and was revealed to be intradural extramedullary spinal metastases (tumors between the spinal cord and the dura mater), rather than schwannoma (Figure 3F). We continued treatment by switching the chemotherapy from alectinib to amrubicin but the patient passed away at the age of 75 years. An autopsy was not performed.

## Discussion

We experienced an extremely rare case of SCLC. Our report has several interesting features. First, an *IAH1-ALK* fusion gene with a novel *ALK* gene rearrangement was identified from the patient’s plasma by a liquid biopsy test. An *ALK* rearrangement is usually found only in NSCLC, and although *ALK* fusion gene-positive SCLC cases have rarely been reported [5-8], some cases responded to alectinib [7]. The disease onset and pathogenesis of the *ALK* fusion gene-positivity in our case are uncertain because we could not assess the pathology of the primary tumor. Considering the discrepancy between the liquid biopsy results and tissue immunostaining, only a small proportion of *ALK* fusion gene-positive cancer cells may have been present in the primary lung lesion. Otherwise, the transformation of *ALK*-positive NSCLC to SCLC might have occurred at the initial stage. Regarding treatment, ALK inhibitors are effective for NSCLC with an *ALK* fusion gene because *ALK* tyrosine kinase is automatically activated by multimerization with fusion partner genes, causing the overexpression of cell proliferation signals [3]. However, standard treatments for SCLC with an *ALK* fusion gene have not been established, although a durable response to ALK inhibitors has been reported [6,7]. Regarding our patient, the primary lesion and metastases continued to progress after treatment with alectinib. Several factors may have contributed to this severe outcome. Liquid biopsy is unable to assess tumors morphologically; thus, the quantity of *ALK*-positive cells in the primary tumor may have been relatively low. If metastasized cancer cells are negative for the *ALK* rearrangement, ALK inhibitors might be ineffective for metastatic lesions. Given the *ALK*-negative results, the effect of alectinib on the right axillary lymph node metastases was expected to be limited. Alternatively, the *IAH1-ALK* fusion gene might not be associated with cancer progression. *ALK* is dimerized and constitutively activated by a coiled-coil oligomerization domain structure produced by the fusion gene [1]. However, IAH1 does not contain this coiled-coil oligomerization domain; therefore, it is assumed that the generated fusion protein may not be activated.

The second feature is that the patient developed intradural extramedullary spinal metastases with myelitis. Primary tumors such as nerve sheath tumors and meningiomas are common pathologies around extramedullary intradural spinal tumors [11]. However, the metastasis rarely presents with the symptoms of longitudinal extensive transverse myelitis [12]. We initially suspected he had only paraneoplastic myelitis because anti-Zic4 antibodies were positive [13,14]. We treated him with high-dose intravenous methylprednisolone. However, the lesion began to expand after treatment. Therefore, the myelitis symptoms in our case may not be caused solely by paraneoplastic mechanisms but may be a combination of metastasis inflammation, tumor infiltration, or vascular compromise from the metastatic tumor. One patient with NSCLC and an *ALK* rearrangement with extramedullary intradural metastases improved with ceritinib treatment [15]. The authors suggested that second-generation ALK inhibitors, such as alectinib and ceritinib, might have good efficacy for metastases of *ALK*-positive NSCLC because of their good permeability through the blood-brain barrier [15]. In our case, the relationship between the *IAH1-ALK* fusion gene and intradural extramedullary spinal metastases with myelitis remains uncertain, because alectinib was not effective against spinal metastases. However, there may have been scope to consider using a different type of ALK inhibitor for our patient.

## Conclusions

In conclusion, an *IAH1-ALK* fusion gene was identified from the plasma of an SCLC patient who developed unusual intradural extramedullary spinal metastasis with myelitis. How the novel *ALK* gene rearrangement influenced the primary lung tumors and metastases in this case remains unclear, and future study of similar cases is required. However, our findings indicated that several types of *ALK* rearrangement may be present in atypical SCLC especially, as well as NSCLC. *ALK* gene rearrangement examination, diagnosis by a multi-disciplinary team including respiratory physicians, neurologists, pathologists, and genetic testing specialists, and proactive treatments including ALK inhibitors may be considered in the future for estimating the prognosis of SCLC patients.
